# The relationship between intracranial artery hemodynamics and subjective cognitive decline in patients with cerebral small vessel disease: a 4D flow study

**DOI:** 10.3389/fnins.2026.1872318

**Published:** 2026-07-01

**Authors:** Hui Sun, Lingjia Xu, Hua Qian

**Affiliations:** 1 Department of Internal Medicine, Shaoxing Second Hospital, The Second Hospital of Shaoxing University, Shaoxing, Zhejiang, China; 2 Department of Neurology, Shaoxing Second Hospital, The Second Hospital of Shaoxing University, Shaoxing, Zhejiang, China; 3 Department of Radiology, The First Affiliated Hospital of Zhejiang Chinese Medical University (Zhejiang Provincial Hospital of Chinese Medicine), Hangzhou, China

**Keywords:** 4D flow MRI, cerebral small vessel disease, hemodynamics, intracranial artery, subjective cognitive decline

## Abstract

**Background:**

Recent studies link disrupted intracranial artery hemodynamics, including pulsatility index (PI), resistance index (RI), and wall shear stress (WSS), to neuroimaging features of cerebral small vessel disease (CSVD). Cognitive dysfunction is a key clinical manifestation of CSVD. Subjective cognitive decline (SCD), considered a pre-mild cognitive impairment stage, enables early identification and intervention to control cognitive decline. Nevertheless, scholarly investigation on SCD in CSVD and its underlying hemodynamic mechanisms remains limited.

**Objective:**

This study aims to utilize 4D flow magnetic resonance imaging (MRI) to explore the effects of intracranial artery hemodynamics on SCD in patients with CSVD.

**Methods:**

This study enrolled 40 patients with CSVD, comprising 20 individuals with SCD and 20 with normal cognition. SCD was evaluated according to established diagnostic criteria using the 9-item Subjective Cognitive Decline Questionnaire (SCD-Q9). Hemodynamic parameters, including PI-flow, PI-area, RI and WSS, were measured in nine major intracranial arteries via 4D flow MRI. Associations between these parameters and cognitive status were examined using logistic regression analysis.

**Results:**

Compared to the cognitively normal group, patients with CSVD and concomitant SCD showed lower arterial elasticity at the C7 segment and the basilar artery (BA), and lower WSS at the C2 segment. Logistic regression analysis further identified abnormal RI-BA was independently associated with SCD in the CSVD cohort.

**Conclusion:**

Altered intracranial artery hemodynamics in patients with CSVD are associated with the presence of SCD. These findings offer mechanistic insight into early cognitive impairment in CSVD and suggest that hemodynamic abnormalities may serve as potential indicators of early cognitive dysfunction in this population.

## 1 Introduction

Cerebral small vessel disease (CSVD) involves pathological changes in the small vessels of the brain, including arteries, veins, and capillaries. Key neuroimaging features of CSVD encompass white matter hyperintensities (WMHs), lacunar infarcts (LI), cerebral microbleeds, enlarged perivascular spaces (EPVS), and brain atrophy (Chojdak-Łukasiewicz et al., 2021). Chronic cerebral vascular pulsatile stress is a proposed pathophysiological mechanism (Luo et al., 2025), and CSVD increases stroke risk and accelerates cognitive decline (Inoue et al., 2023). Subjective cognitive decline (SCD) describes an individual’s self-reported experience of worsening cognitive function in the absence of objective impairment on formal neuropsychological testing (Lin et al., 2019). SCD is generally considered a precursor to mild cognitive impairment (MCI). Investigating SCD in the context of CSVD offers a means to identify cognitive deterioration at an earlier stage, which holds important implications for reducing CSVD-related dementia and improving patients’ quality of life. Nevertheless, studies examining the cerebral hemodynamic mechanisms linking CSVD to SCD remain scarce.

Current research frequently explores the relationship between hemodynamic parameters and the neuroimaging markers of CSVD. The pulsatility index (PI) measures arterial pulsatility. The resistance index (RI) evaluates vascular resistance, serving as an indicator of stenosis severity and the condition of the distal microvascular bed (Rivera-Rivera et al., 2016). Clinically, both PI and RI are utilized to assess vascular stenosis, obstruction, and perfusion, making them essential metrics for gauging pulsatility and stenosis in the diagnosis and monitoring of cardiovascular and cerebrovascular diseases. Recent work suggests that increased cerebrovascular pulsatility caused by arterial stiffness in distal cerebral arteries and microvasculature is linked to CSVD pathogenesis and cerebral clearance mechanisms (Kamimura et al., 2023; Riba-Llena et al., 2018). Reduced wall shear stress (WSS) promotes endothelial dysfunction and vascular remodeling (Soulat et al., 2020), acting as a hemodynamic biomarker of atherosclerosis. In conjunction with systemic vascular risk factors, low WSS contributes to the formation of atherosclerotic plaques and fibro-inflammatory lipid accumulation, which can impair cerebral perfusion (Cunningham and Gotlieb, 2005) and elevate the risk of CSVD (Liu et al., 2016).

Hemodynamic alterations are central to CSVD pathophysiology and closely linked to both structural progression and cognitive decline. Enhancing cerebral circulatory function represents a promising therapeutic target in CSVD. Nevertheless, their specific role in early cognitive impairment, particularly in the stage of SCD, remains insufficiently examined. Recent advances show that 4D flow MRI enables non-invasive assessment of intracranial hemodynamics in large vessels, potentially identifying patients amenable to early intervention. Here, we used 4D flow MRI to quantify key hemodynamic parameters and assess their association with SCD in CSVD patients.

## 2 Materials and methods

### 2.1 Participants

A total of 40 patients were included in this study, with 20 patients in the CSVD combined with SCD group (CSVD-SCD group) and 20 patients in the CSVD with normal cognition group (CSVD-NC group). Inclusion criteria were: (1) age ≥ 45 years; (2) fulfillment of neuroimaging diagnostic criteria for CSVD (Duering et al., 2023); (3) availability of complete clinical and imaging data. Exclusion criteria comprised: (1) vascular occlusion, severe stenosis, or developmental anomalies that could confound hemodynamic assessment; (2) history of traumatic brain injury, brain tumor, stroke, other major neurological or psychiatric disorders; (3) other serious systemic illnesses; (4) contraindications to or inability to tolerate MRI.

### 2.2 Clinical data

Two board-certified neurologists assessed cognitive state using the validated 9-item SCD Questionnaire (SCD-Q9) and applied standard diagnostic criteria (Gifford et al., 2015). The SCD diagnostic criteria: (1) it must be self-reported, with a continuous decline in cognitive ability compared to the previous normal state, and unrelated to acute events; (2) within the normal age, gender, education level range, and have normal standard cognitive test levels, not reaching the cognitive impairment degree of MCI, assessed by Mini-Mental State Examination (MMSE). Exclusion criteria include: (1) MCI or dementia; (2) not attributable to mental disorders or neurological diseases and other diseases, drugs, or substance abuse (Gifford et al., 2015). Trained researchers collected demographic data and medical history including age, sex, height, weight, year of education, smoking and drinking history, hypertension, diabetes, hyperlipidemia and coronary disease via face-to-face interviews. Sleep quality over the prior month was evaluated with the Pittsburgh Sleep Quality Index (PSQI), scores ≥ 7 indicated sleep disturbance (Gao et al., 2024). Depressive and anxiety symptoms were assessed using the Patient Health Questionnaire-9 (PHQ-9) and Generalized Anxiety Disorder-7 (GAD-7), respectively, scores ≥ 5 on either scale indicated clinically relevant symptoms (Fortini et al., 2024).

### 2.3 Image processing and analysis

#### 2.3.1 MRI acquisition

All patients underwent MRI on a 3.0T GE SIGNA Premier scanner with a 48-channel head coil. We acquired whole-brain, time-resolved 4D flow data using a three-dimensional radial under-sampled sequence. Imaging parameters: velocity encoding = 120 cm/s; FOV = 24 × 24 cm^2^; TR/TE = 4.5/2.4 ms; isotropic spatial resolution = 1.2 mm; flip angle = 12°; scan time ≈ 10 min. Concurrent photoplethysmography via a GE pulse oximeter provided cardiac timing signals. Using these signals and the 4D flow data, we reconstructed cardiac-resolved velocity, amplitude, and angiographic images across 24 phases. Amplitude and angiographic images were visually inspected for motion-related vascular blurring; minor corrections were applied when needed. Background phase offset and velocity aliasing were corrected.

Structural scans included: T1-weighted (TR/TE = 1750/24 ms; FOV = 240 × 240 mm^2^; voxel size = 0.8 × 0.9 × 5.0 mm^3^), T2-weighted (TR/TE = 5457/100 ms; FOV = 240 × 240 mm^2^; voxel size = 0.8 × 0.8 × 5 mm^3^), T2-Fluid-Attenuated Inversion Recovery (T2-FLAIR) (TR/TE = 9000/100 ms; FOV = 240 × 240 mm^2^; voxel size = 0.8 × 1.2 × 5.0 mm^3^), and Susceptibility-weighted imaging (TR/TE = 38.3/23.3 ms; FOV 240 × 240 mm^2^; voxel size 0.5 × 0.5 × 1 mm^3^). Diffusion weighted imaging used single-shell acquisition (*b* = 1000 s/mm^2^, 3 directions) with spin-echo EPI (TR/TE = 2509/75.8 ms; FOV = 240 × 240 mm^2^; voxel size = 1.9 × 1.9 × 5.0 mm^3^; phase encoding = posterior−anterior), plus a matched b0 image with reversed phase encoding to correct EPI distortion.

#### 2.3.2 Evaluation of CSVD

**FIGURE 1:**
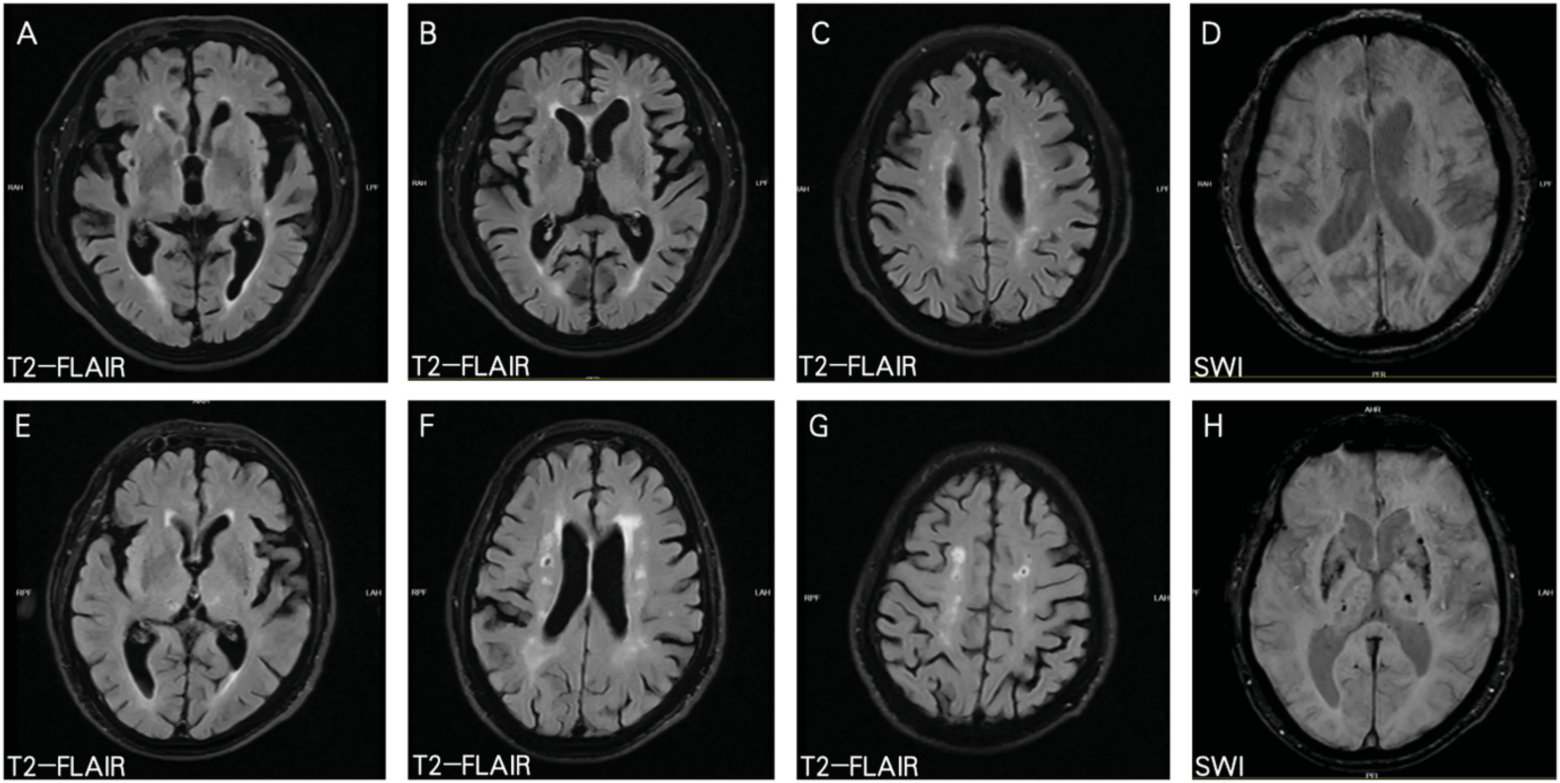
Diagram of cerebral small vessel disease (CSVD) evaluation. Case 1 **(A–D)**, LI in the right basal ganglia region **(A)**; WMH grade level 1 **(A,B)**; total sum of EPVS in the basal ganglia area ≥ 10 **(A,B)**; no microbleeds **(D)**; total burden of CSVD is 2. Case 2 **(E,F)**, WMH grade level 2 **(E,F)**; total sum of EPVS in the basal ganglia area ≥ 10 **(E)**; LI in in the right lateral ventricle and the left parahippocampal region **(F,G)**; microbleeds in the thalamus **(H)**; total burden of CSVD is 3.

Two experienced researchers independently evaluated the structural MRI scans of each patient according to established CSVD criteria (Duering et al., 2023). WMHs were graded using the modified Fazekas scale for periventricular and deep white matter regions (Fazekas et al., 1987). EPVS were assessed on T2-FLAIR imaging in the basal ganglia (BG) and centrum semiovale (CSO) (Doubal et al., 2010). Cerebral microbleeds were identified and counted on susceptibility-weighted imaging, with deep and lobar microbleeds recorded separately. LI were identified on T2-FLAIR imaging. The overall CSVD burden was assessed using Wardlaw’s 4-point scale: one point each for (1) periventricular WMHs ≥ 3 or deep WMHs ≥ 2; (2) combined BG and CSO EPVS ≥ 10; (3) ≥1 deep microbleed; and (4) ≥1 lacune. The sum yielded the total CSVD score (Staals et al., 2014). Representative imaging examples are shown in Figure 1.

#### 2.3.3 Post-processing of 4D flow MRI

**FIGURE 2:**
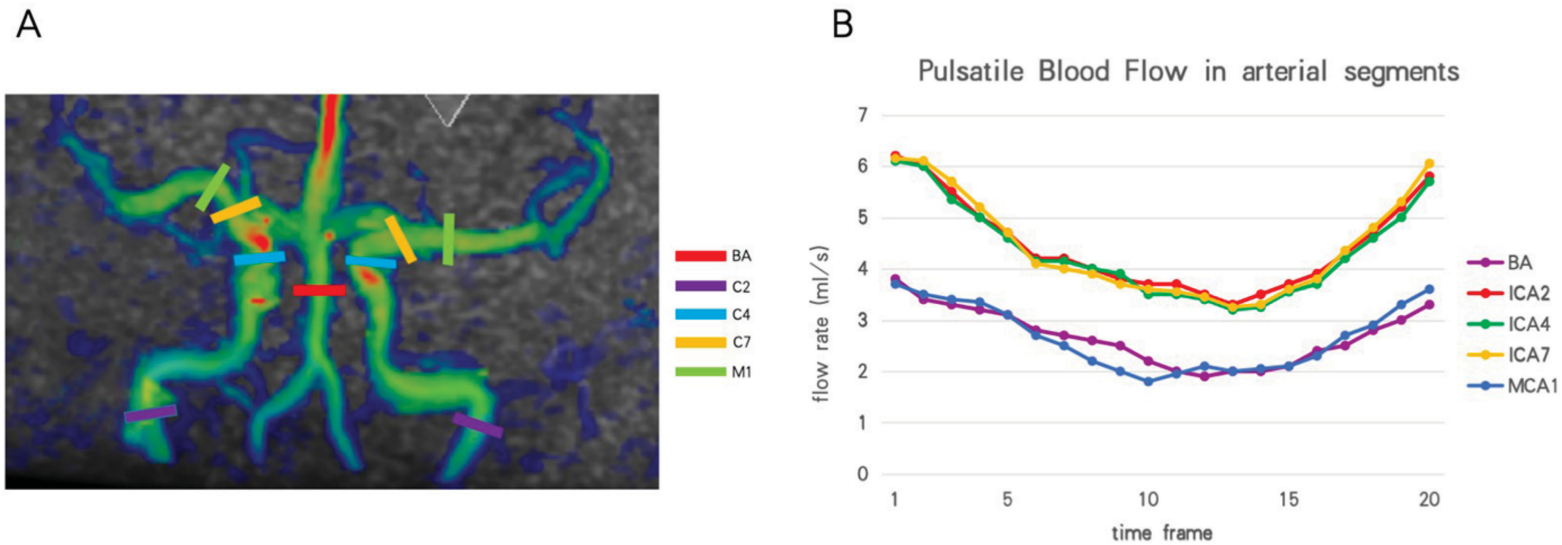
Selected arterial segments and pulsatile blood flow through the cardiac cycle. **(A)** Nine vessel segments placed with planes perpendicular to the vessel orientations from a representative individual. **(B)** Pulsatile flow waveforms of selected vessel segments through the cardiac cycle from a representative individual. BA, basilar artery; C2, petrous segment of internal carotid artery; C4, cavernous segment of internal carotid artery; C7, ophthalmic segment of internal carotid artery; M1, origin of middle cerebral artery; ICA, internal carotid artery; MCA, middle cerebral artery.

We analyzed 4D flow data using CVI42 software (version 6.3.1, Circle Cardiovascular Imaging Inc.,) (Xie et al., 2024). In order to eliminate the influence of hemodynamic effects caused by physiological vascular distortion and to ensure the feasibility of the calculation of PI-area, nine straight arterial segments with large lumens were selected based on lumen size and minimal pulsatility artifact: bilateral internal carotid arteries (ICA) including petrous (C2), cavernous (C4) and ophthalmic (C7) segments; bilateral middle cerebral artery (MCA) origins, and the mid-basilar artery (BA) (Figure 2). For each vessel, we placed three equally spaced cross-sectional planes. Preprocessing included anti-aliasing and displacement correction to enable automated centerline tracking and vascular segmentation. Hemodynamic parameters were computed per plane and averaged across the three planes and both sides. These included: cross-sectional area (A); mean and peak blood flow velocity (Qmean, Qpeak); PI-flow = (Qmax − Qmin)/Qmean; PI-area = (Amax − Amin)/Amean); RI = (Qmax − Qmin)/Qmax; and WSS. All values represent intra-cardiac-cycle maxima, minima, or means.

Cohen’s kappa value was used to assess the consistency between the results of two researchers. Disagreements are resolved through discussion.

### 2.4 Statistic analysis

Statistical analysis was performed using Statistical Package for the Social Sciences (SPSS) software (version 27.0, IBM SPSS Inc., Chicago, IL, USA). Given the small sample size (*n* = 40), normality was assessed with the Shapiro-Wilk test. Continuous variables were reported as mean ± SD if normally distributed, or median (interquartile range) if skewed. The inter-group differences of quantitative variables were analyzed using the independent two-sample *t*-test for parametric data and the Mann-Whitney U test for non-parametric data. Categorical variables were compared using chi-square tests.

Pearson correlation was used for continuous hemodynamic parameters; point-biserial correlation was applied for binary outcomes. Multivariable logistic regression modeled cognitive state in CSVD patients as the outcome. All factors were Z-score standardized. Model 1 included each hemodynamic parameter individually; Model 2 adjusted for age, sex, and year of education; Model 3 further adjusted for BMI, hypertension, diabetes, hyperlipidemia, coronary disease, smoking, alcohol use, sleep disturbance, depressive and anxiety symptoms. All multivariate regression analyses and multiple comparisons of hemodynamic parameters were performed for exploratory purposes, no multiple comparison correction was applied. Results are presented as odds ratios (OR) with 95% confidence intervals (95% CI). Statistical significance was set at *p* < 0.05 (two-tailed).

## 3 Results

### 3.1 Participant characteristics

Our study included 40 patients with CSVD, among whom 20 had normal cognition and 20 had SCD. The incidence of hypertension and diabetes in the CSVD-NC group was higher (χ^2^ = 7.62, *p* = 0.006, χ^2^ = 4.29, *p* = 0.038). The total score of CSVD burden in the CSVD-SCD group was higher, but the difference was not statistically significant (*U* = 164.5, *p* = 0.301). No significant differences were found in other demographic indicators. The details are shown in Table 1 (Demographics data). There were good agreements between the two researchers in the assessments of CSVD and hemodynamic measurements (Kappa = 0.672 and 0.634).

**TABLE 1:** Participants characteristics.

Variable	CSVD-NC	CSVD-SCD	Effect size	*p*
Demographics data				
Male	12 (60%)	10 (50%)	0.40	0.525
Age	62.5 (6.6)	60.9 (6.7)	0.76	0.451
BMI	25.1 (5.9)	25.1 (5.3)	0.03	0.978
Year of education	10 (6, 11)	10 (5, 12)	63.5	0.634
Smoking history	8 (40%)	7 (35%)	0.11	0.744
Drinking history	6 (30%)	7 (35%)	0.11	0.736
Hypertension	18 (90%)	10 (50%)	7.62	0.006
Diabetes	9 (45%)	3 (15%)	4.29	0.038
Hyperlipidemia	7 (35%)	8 (40%)	0.11	0.744
Coronary disease	5 (25%)	3 (15%)	–	0.695
Sleep disturbance	11 (55%)	9 (45%)	0.40	0.527
Depressive symptoms	5 (25%)	6 (30%)	0.13	0.723
Anxiety symptoms	7 (35%)	9 (45%)	0.42	0.519
CSVD burden	1 (1, 2)	1 (1, 3)	164.5	0.301
Hemodynamic measurements				
PI-flow-C2	0.70 (0.13)	0.72 (0.13)	0.54	0.592
PI-flow-C4	0.69 (0.11)	0.69 (0.11)	0.05	0.965
PI-flow-C7	0.63 (0.07)	0.70 (0.05)	3.68	**<0.001**
PI-flow-M1	0.61 (0.08)	0.64 (0.09)	1.07	0.291
PI-flow-BA	0.71 (0.15)	0.70 (0.16)	0.29	0.774
PI-area-C2	0.100 (0.007)	0.101 (0.007)	0.39	0.697
PI-area-C4	0.090 (0.006)	0.092 (0.010)	0.47	0.640
PI-area-C7	0.090 (0.008)	0.091 (0.009)	0.33	0.746
PI-area-M1	0.087 (0.006)	0.088 (0.008)	0.24	0.812
PI-area-BA	0.093 (0.010)	0.094 (0.012)	0.09	0.932
RI-C2	0.60 (0.08)	0.61 (0.08)	0.31	0.756
RI-C4	0.62 (0.09)	0.63 (0.09)	0.27	0.792
RI-C7	0.65 (0.10)	0.66 (0.10)	0.28	0.778
RI-M1	0.63 (0.11)	0.64 (0.11)	0.26	0.799
RI-BA	0.54 (0.15)	0.63 (0.09)	2.22	**0.033**
WSS-C2	0.43 (0.08)	0.38 (0.05)	2.46	**0.019**
WSS-C4	0.43 (0.07)	0.43 (0.06)	0.19	0.849
WSS-C7	0.43 (0.10)	0.41 (0.07)	0.77	0.447
WSS-M1	0.42 (0.10)	0.40 (0.08)	0.55	0.588
WSS-BA	0.41 (0.10)	0.41 (0.09)	0.08	0.935

CSVD, cerebral small vessel disease; NC, normal cognition; SCD, subjective cognitive decline; BMI, body mass index; PI, pulsatile index; RI, resistance index; WSS, wall shear stress; C2, petrous segment of internal carotid artery; C4, cavernous segment of internal carotid artery; C7, ophthalmic segment of internal carotid artery; M1, origin of middle cerebral artery; BA, basilar artery. Bold values mean “*p* < 0.05”.

### 3.2 Intergroup analysis and correlation analysis of hemodynamic measurements

Compared with the CSVD-NC group, the CSVD-SCD group showed higher PIflow-C7 and RI-BA (*F* = 3.68, *p* < 0.001; *F* = 2.22, *p* = 0.033). Although PI-area did not differ significantly between groups, values tended to be higher across all vessel segments in the CSVD-SCD group (all *p* > 0.05). Similarly, RI was consistently higher across all segments in the CSVD-SCD group (*p* < 0.05 for RI-BA; *p* > 0.05 for others).

**FIGURE 3:**
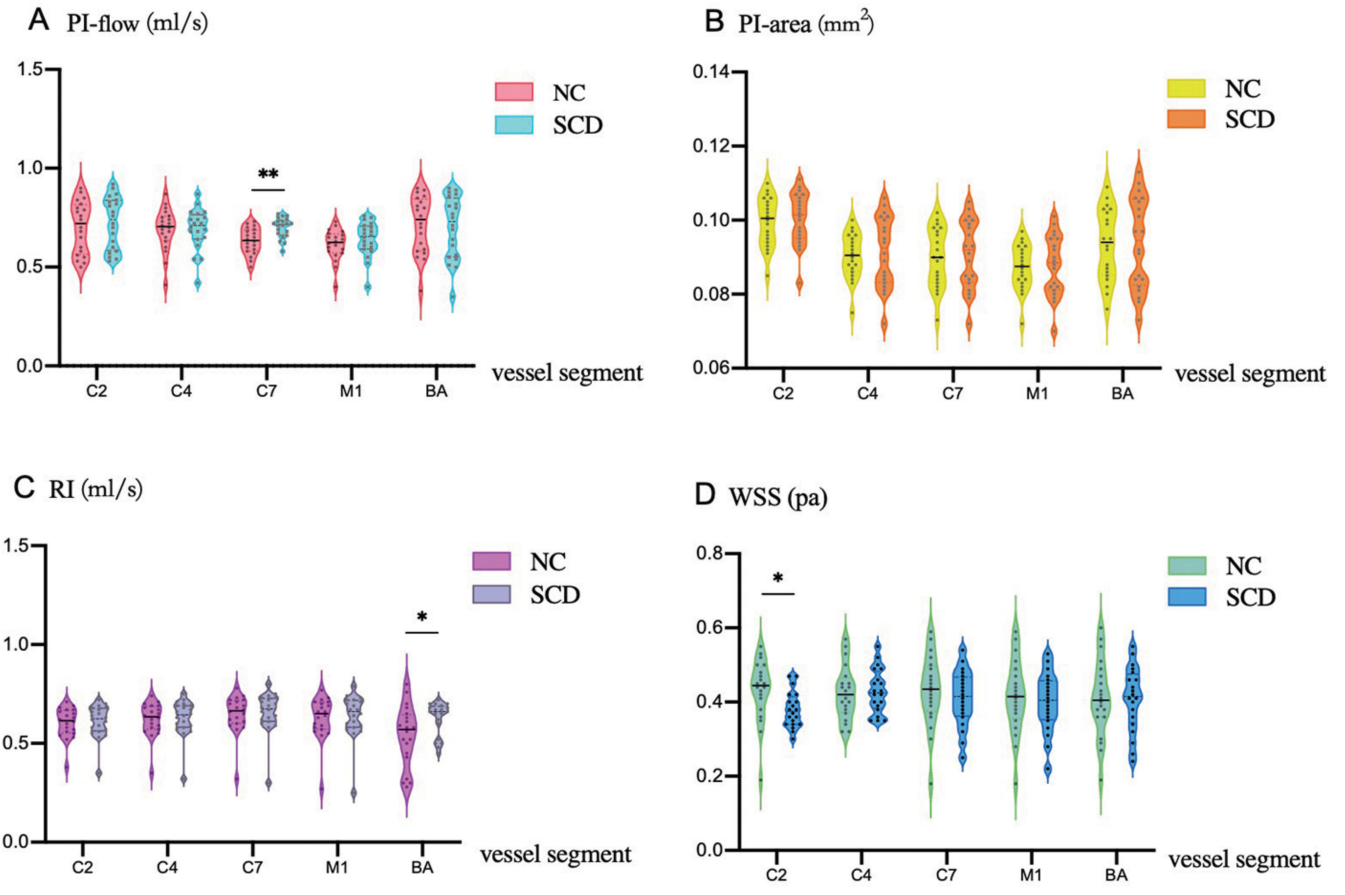
Intergroup comparisons of PI-flow, PI-area, RI and WSS between CSVD-NC group and CSVD-SCD group. **(A)** Intergroup comparison between the two groups in PI-flow. **(B)** Intergroup comparison between the two groups in the PI-area. **(C)** Intergroup comparison between the two groups in RI. **(D)** Intergroup comparison between the two groups in WSS. PI, pulsatility index; RI, resistance index; WSS, wall shear stress; C2, petrous segment of internal carotid artery; C4, cavernous segment of internal carotid artery; C7, ophthalmic segment of internal carotid artery; M1, origin of middle cerebral artery; BA, basilar artery; SCD, subjective cognitive decline; NC, normal cognition; *p < 0.05; **p < 0.001.

In contrast, WSS-C2 was lower in the CSVD-SCD group versus the CSVD-NC group (*F* = 2.46, *p* = 0.019). While no other WSS differences reached significance, WSS-C2, WSS-C7, and WSS-M1 were generally lower in the CSVD-SCD group (*p* < 0.05 for WSS-C2; *p* > 0.05 for others). The above results are shown in Table 1 (Hemodynamic measurements), Figure 3.

Further correlation analysis revealed that the values of PI-flow-C7, RI-BA, and WSS-C2 were correlated with whether CSVD was combined with SCD (*r* = 0.513, *p* < 0.001; *r* = 0.014, *p* = 0.033; *r* = −0.426, *p* = 0.006).

### 3.3 Multivariate regression analysis of hemodynamic measurements and SCD in CSVD

Multivariable logistic regression assessed whether hemodynamic parameters from each vascular segment were independently associated with the cognitive state in CSVD patients. For each segment, PI-flow, PI-area, RI, and WSS were entered separately as influencing factors. Models were adjusted for age, sex, BMI, education years, vascular risk factors (hypertension, diabetes, hyperlipidemia, coronary disease), lifestyle habits (smoking, alcohol use) and emotional state (sleep disturbance, depressive and anxiety symptoms).

As shown in Table 2, in Model 1 (unadjusted), abnormal PI-flow-C7 (OR = 0.26, *p* = 0.004), RI-BA (OR = 0.46, *p* = 0.044), and WSS-C2 (OR = 1.09, *p* = 0.028) were significantly associated with the cognitive state in CSVD patients. In Model 2 (adjusted for age, sex, year of education), associations strengthened for PI-flow-C7 (OR = 0.14, *p* = 0.002), RI-BA (OR = 0.42, *p* = 0.034, WSS-C2 (OR = 1.15, *p* = 0.018), and WSS-BA (OR = 0.56, *p* = 0.005). In Model 3 (fully adjusted), only RI-BA remained significant (OR = 0.05, *p* = 0.029), indicating that higher value of RI-BA independently associated with greater likelihood of SCD in CSVD.

**TABLE 2:** Multiple regression of hemodynamic measurements and cognitive state in CSVD.

Variables	Model 1		Model 2		Model 3	
	OR (95% CI)	*p*	OR (95% CI)	*p*	OR (95% CI)	*p*
PI-flow-C2	0.84 (0.45 ∼ 1.57)	0.581	0.80 (0.39 ∼ 1.63)	0.541	1.58 (0.41 ∼ 6.10)	0.511
PI-flow-C4	0.99 (0.53 ∼ 1.85)	0.963	0.95 (0.48 ∼ 1.87)	0.881	1.54 (0.50 ∼ 4.72)	0.450
PI-flow-C7	0.26 (0.10 ∼ 0.65)	**0.004**	0.14 (0.04 ∼ 0.47)	**0.002**	0.00 (0.00 ∼ 1.20)	0.057
PI-flow-M1	0.70 (0.36 ∼ 1.36)	0.289	0.65 (0.30 ∼ 1.39)	0.267	0.54 (0.17 ∼ 1.66)	0.280
PI-flow-BA	1.10 (0.59 ∼ 2.06)	0.767	1.10 (0.54 ∼ 2.24)	0.797	2.60 (0.65 ∼ 10.40)	0.176
PI-area-C2	0.88 (0.47 ∼ 1.65)	0.689	0.82 (0.40 ∼ 1.67)	0.584	1.62 (0.48 ∼ 5.43)	0.434
PI-area-C4	0.86 (0.45 ∼ 1.61)	0.629	0.78 (0.38 ∼ 1.59)	0.495	1.29 (0.41 ∼ 4.03)	0.667
PI-area-C7	0.90 (0.48 ∼ 1.69)	0.738	0.85 (0.42 ∼ 1.72)	0.650	1.61 (0.47 ∼ 5.46)	0.448
PI-area-M1	0.92 (0.49 ∼ 1.73)	0.806	0.86 (0.42 ∼ 1.74)	0.670	1.55 (0.48 ∼ 5.02)	0.466
PI-area-BA	0.97 (0.52 ∼ 1.82)	0.930	0.94 (0.47 ∼ 1.90)	0.865	1.93 (0.51 ∼ 7.25)	0.331
RI-C2	0.90 (0.48 ∼ 1.70)	0.749	0.82 (0.41 ∼ 1.67)	0.589	1.51 (0.50 ∼ 4.55)	0.464
RI-C4	0.92 (0.49 ∼ 1.72)	0.786	0.82 (0.41 ∼ 1.67)	0.594	1.48 (0.51 ∼ 4.35)	0.471
RI-C7	0.91 (0.48 ∼ 1.71)	0.772	0.80 (0.39 ∼ 1.64)	0.545	1.41 (0.49 ∼ 4.05)	0.528
RI–M1	0.92 (0.49 ∼ 1.73)	0.793	0.82 (0.40 ∼ 1.66)	0.578	1.41 (0.49 ∼ 4.03)	0.520
RI-BA	0.46 (0.22 ∼ 0.98)	**0.044**	0.42 (0.19 ∼ 0.94)	**0.034**	0.05 (0.00 ∼ 0.73)	**0.029**
WSS-C2	1.09 (1.01 ∼ 1.18)	**0.028**	1.15 (1.03 ∼ 1.30)	**0.018**	1.33 (0.98 ∼ 1.81)	0.067
WSS-C4	0.99 (0.93 ∼ 1.06)	0.845	0.86 (0.72 ∼ 1.01)	0.069	0.69 (0.47 ∼ 1.02)	0.063
WSS-C7	1.03 (0.96 ∼ 1.09)	0.438	1.04 (0.77 ∼ 1.39)	0.811	0.90 (0.57 ∼ 1.40)	0.628
WSS-M1	1.02 (0.96 ∼ 1.08)	0.578	0.88 (0.64 ∼ 1.19)	0.400	0.66 (0.34 ∼ 1.25)	0.201
WSS-BA	1.00 (0.94 ∼ 1.07)	0.932	0.56 (0.37 ∼ 0.84)	**0.005**	0.30 (0.09 ∼ 1.03)	0.056

Model 1: univariate regression model. Model 2: multivariable regression model, controlled for age, sex, year of education. Model 3: multivariable regression model, controlled for age, sex, year of education, BMI, hypertension, diabetes, hyperlipidemia, coronary disease, smoke history, drink history, sleep disturbance, depressive symptoms, anxiety symptoms. PI, pulsatile index; RI, resistance index; WSS, wall shear stress; C2, petrous segment of internal carotid artery; C4, cavernous segment of internal carotid artery; C7, ophthalmic segment of internal carotid artery; M1, origin of middle cerebral artery; BA, basilar artery. Bold values mean “*p* < 0.05”.

## 4 Discussion

Cerebral small vessel disease is a progressive microvascular disorder that significantly affects brain health in the aging population (Cannistraro et al., 2019; Hainsworth et al., 2024; Wardlaw et al., 2019). Although its clinical manifestations are varied, accumulating evidence indicates that CSVD disrupts the function of the neurovascular unit and the integrity of the microcirculation, contributing to vascular cognitive impairment (Hua et al., 2023). The new research further emphasizes the correlation between hemodynamic dysfunction (particularly in large intracranial blood vessels) and the progression of CSVD (Liu et al., 2025; Luo et al., 2025).

Cerebral small vessel disease is characterized by abnormal PI and WSS in both arterial and venous vessels across multiple brain regions (Luo et al., 2025). Global cerebral blood flow declines as the disease advances irrespective of lesion location. This reduction in perfusion leads to decreased mitochondrial density, lower ATP content, and diminished neuronal activity. These hemodynamic disturbances contribute to microcirculatory dysfunction and heightened blood–brain barrier permeability (Erdener and Dalkara, 2019; Luo et al., 2025; Wardlaw et al., 2013), which further impair fluid transport across the barrier and through perivascular spaces, thereby promoting progressive brain injury (Goldsmith, 2022). Elevated PI and decreased WSS reflect underlying vascular endothelial dysfunction and impaired cerebral autoregulation, processes central to the pathophysiology of CSVD.

In this study, we applied 4D flow MRI to characterize cerebral arterial hemodynamics in patients with CSVD across different cognitive states, in order to clarify how cerebral blood flow affects early cognitive decline within this population. Unlike normal aging, Alzheimer’s disease (AD) is marked by region-specific cerebral blood flow alterations that can precede cognitive symptoms (Mokhber et al., 2021). Our findings demonstrated that, compared to the CSVD-NC group, the CSVD-SCD group exhibited significantly higher PI-flow at the C7 segment and higher RI in BA segment, along with significantly lower WSS at C2 segment. Correlation analyses further associated abnormal PI-flow-C7, RI-BA, and WSS-C2 with the presence of SCD in CSVD. Multivariable logistic regression identified RI-BA as an independent impaired factor of SCD, suggesting that elevated RI in the BA segment is linked to a greater likelihood of early cognitive symptoms. These findings are consistent with prior research. Zhang et al. (2021) similarly reported that reduced cerebral blood flow correlates with cognitive worsening and accelerated decline. Cao et al. (2025) observed moderate positive correlations between cerebral blood flow, velocity parameters, and cognitive scores, noting significantly lower flow metrics in CSVD patients with cognitive impairment. Furthermore, Rivera-Rivera et al. (2016) documented markedly reduced mean flow across cerebral arterial segments in patients with MCI, which aligns with the hemodynamic patterns observed in our study.

Recent studies suggest that arterial stiffness increases pulsatility in distal cerebral arteries and the microvascular network, tying it to the onset and progression of CSVD (Birnefeld et al., 2020; Jochemsen et al., 2015). The elevated artery pulsation is also associated with cognitive impairment and may help predict dementia in individuals with SCD or MCI (Chung et al., 2017). Moreover, the PI has been found to correlate with markers of glymphatic clearance, a process critically involved in the pathogenesis of AD (Xie et al., 2024). Wu et al. (2024) first reported that in patients with CSVD, an elevated PI is independently associated with cognitive impairment and greater total CSVD burden. Specifically, PI demonstrates a significant negative correlation with cognitive performance and a positive correlation with overall disease severity. These findings are consistent with prior observations that elevated PI reflects underlying cerebral hypoperfusion and microvascular injury, and is related to cognitive decline and increased dementia risk (Chung et al., 2017). Elevated PI has also been validated as an indicator of dementia and executive dysfunction in CSVD (Lorenzini et al., 2024). RI, a parameter reflecting downstream vascular resistance, provides complementary information about the distal microvascular bed. Although mathematically related to PI, RI specifically conveys information about vascular stenosis, perfusion status, and microvascular impedance (Rivera-Rivera et al., 2016). RI was identified to be associated with cognitive impairment and WMHs (Pahlavian et al., 2021). Elevated RI has likewise been significantly associated with cognitive decline (Shi et al., 2018), which aligns with the results of the present study.

Wall shear stress describes the frictional force generated by blood flow along the vessel wall. Reduced or oscillatory WSS is known to related to local endothelial dysfunction and the progression of atherosclerosis (Sluimer, 2024; Thondapu et al., 2017). Liu et al. (2016) reported that, after adjusting for confounders, both average and peak carotid WSS were independently associated WMHs and lower MMSE scores. This suggests that disturbed local hemodynamic forces are associated with white matter injury and cognitive decline in older adults. Our results indicate that WSS in the C2 segment is lower in CSVD patients with SCD. Decreased WSS in the ICA and BA has previously been linked to MCI and AD (van Es et al., 2009). For instance, individuals with MCI or AD exhibit significantly lower carotid WSS compared to individuals with normal cognition (van Es et al., 2009). As a central hemodynamic factor, WSS is increasingly recognized as an important harmful factor to the pathophysiology of chronic cerebrovascular disorders (Chen et al., 2019; Mazzi et al., 2020).

This study has several limitations. First, the sample size is relatively small and recruited from a single center without a healthy control group, mainly due to budgetary constraints. The fully adjusted regression containing numerous covariates may cause overfitting and unstable odds ratio estimation. Besides, multiple uncorrected comparisons increase the possibility of false-positive outcomes. As an exploratory analysis, relevant results should be interpreted prudentially, and larger sample cohorts are required to confirm our conclusions. The limited sample size also prevented stratified analyses based on CSVD subtypes or regional cerebral blood flow patterns. Second, laboratory data on vascular risk factors were incomplete. For example, although a history of hyperlipidemia was recorded, specific lipid measures such as total cholesterol and low-density lipoprotein levels were not consistently available, which may affect a full assessment of vascular risk. Third, technical limitations of 4D flow MRI should be acknowledged. Due to constraints in temporal and spatial resolution, signal loss can occur in smaller vessels with slower flow. Our analysis was therefore restricted to larger arterial segments, which may influence the accuracy of hemodynamic measurements. Finally, the cross- sectional design of this study does not allow causal inferences. Longitudinal studies will be necessary to confirm these observations and to clarify the temporal relationship between hemodynamic changes and cognitive outcomes in CSVD.

## 5 Conclusion

This study demonstrates that 4D flow MRI can be effectively used to assess hemodynamic parameters in the major cerebral arteries of patients with CSVD and their association with SCD. Our findings establish a link between altered cerebral hemodynamics and very early cognitive impairment in this population. These results could help identify individuals at risk of early cognitive decline who may benefit from interventions designed to improve cerebral circulation. Moreover, this work points toward a potential direction for developing targeted clinical strategies to preserve cognitive function in patients with CSVD.

## Data Availability

The raw data supporting the conclusions of this article will be made available by the authors, without undue reservation.
